# Improving rowing performance by adjusting oar blade size and angle

**DOI:** 10.3389/fspor.2023.1109494

**Published:** 2023-03-09

**Authors:** W. C. A. M. van Nieuwburg, B. J. J. van Spreuwel, M. T. K. Tran, M. D. Yang, A. Greidanus, G. Mulder, M. J. Tummers, J. Westerweel, W. Suijker, R. van Wijk

**Affiliations:** ^1^ Laboratory for Aero & Hydrodynamics, Delft University of Technology, Delft, Netherlands; ^2^ Maritime Research Institute Netherlands (MARIN), Wageningen, Netherlands

**Keywords:** rowing blade, performance, efficiency, optimisation, robot

## Abstract

The principal aim of the work presented here is to investigate and demonstrate that a forward tilted rowing blade would result in a more efficient and effective motion of the blade through the water that would result in a higher boat speed when an equal input power is provided. A 1:5 scaled rowing boat is used to determine the performance of rowing blades with different sizes and blade angles. This is used to validate the results of a previous study where the optimal blade angle of 15${}^{\circ }$ with respect to the oar shaft was determined ( 1). The input power and speed of the rowing boat can be compared between original and modified oar blades. Measurements in a towing tank demonstrate that a modified rowing blade result in faster rowing by 0.4% at the same input power. Maintaining the same stroke rate, the improvement of the blade efficiency is compensated by using a 4–6% increased blade area to yield the same input power.

## Introduction

Competitive rowing has been a part of the modern Olympic games since the 1900 Paris Olympics, in which the Dutch rowers Brandt and Klein won gold in the men’s coxed pair in a time of 7:34.20. The current world’s best time in the same discipline is 6:33.26, set in 2014. This large improvement in time can be attributed to significant improvements in both training methods and boat technology; important technological developments are the introduction of the sliding seat, light weight hydrodynamic construction, the use of outriggers, and improved materials (2).

**Figure 1:**
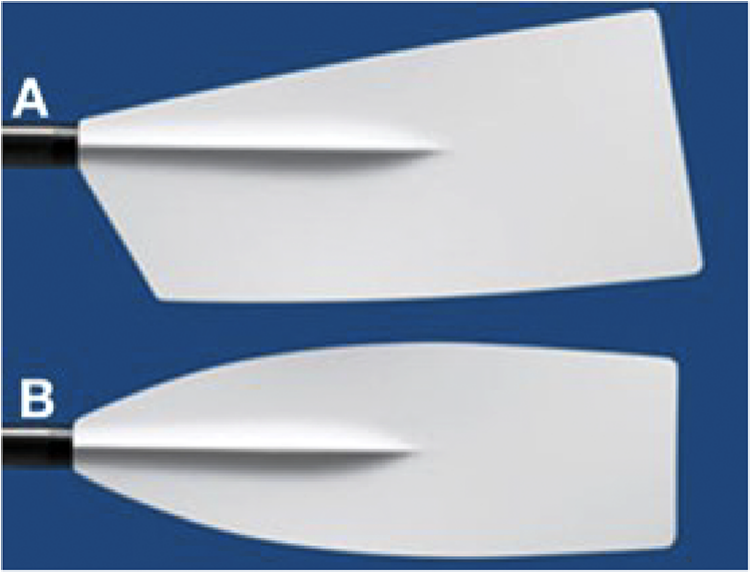
Different rowing blades: (**A**) Big blade, (**B**) Macon blade. From: www.concept2.nl/en/oars/oar-options/blades

The oars have changed over time as well. The largest difference in oar blades is shown in Figure 1. The “Macon” oar blade was used until the 90s when the “Big blade” was introduced. The improved design of this blade was made possible because of advancement in materials. More recently more advanced blade types have been introduced, like the “Smoothie 2” and the “Comp” blades, that are now commonly used in competitive rowing. Nonetheless, a lot of research was done on the Big Blade (1, 3–7), and therefore the same blade is selected in the present investigation.

Extensive field measurements have been carried out by Kleshnev (8), with a strong focus on the biomechanics of rowing. Improvements in rowing can also be attributed to better understanding of the fundamentals of hydrodynamics. The rowing motion is an unsteady and complex motion. The “drive phase” begins when the blade is lowered into the water, and then moves through the water as the rower pulls on the oar, until the blade is lifted from the water. During the drive phase the athlete provides the forward propulsion. The drive phase produces a complex flow around the oar blade, including motion of the water-air interface, which makes it difficult to predict the hydrodynamic forces acting on the blade using computational fluid dynamics.

Instead, detailed experiments provide insight in the hydrodynamics of the blade. A recent investigation by Grift et al. (1) suggests that a forward angled oar blade would improve the efficiency of the rowing blade action. Summarizing their findings, an optimal angle of $\beta =15^{\circ }$ was found that would result in a 20% increase in propulsion efficiency compared to the standard oar blade at $\beta =0^{\circ }$ angle. The forward angled blade causes the total impulse, defined as the time-integrated hydrodynamic force on the blade in the water during the driving phase, to be directed parallel to the boat, whereas for the conventional blade (with $\beta =0^{\circ }$) the impulse is directed outward at an angle of about $15^{\circ }$. Clearly, the component of the impulse that is normal to the direction in which the boat moves, implies a loss. This can be observed in practice from the motion of the eddies generated during the driving phase that clearly move at an outward angle with respect to the boat. However, the blade angle that gives rise to the maximum propulsion efficiency does not coincide with the blade angle that produces maximum effectiveness (defined as the time integral of the propulsive force component in the boat velocity direction) that occurs near 0${}^{\circ }$ blade angle. This is directly related to differences in the instantaneous flow fields around the blades for the two blade angles, especially regarding the differences in behaviour of the leading edge vortices that are formed during the initial phase of the stroke cycle; see Figures 14 and 23 in the paper by Grift et al. (1). It is conjectured that tilting the blade to align the impulse vector of the hydrodynamic force parallel to the direction of the moving boat also applies to other blade designs, albeit that the suggested optimal angle may be different than the 15${}^{\circ }$ angle that was found for the Big Blade.

In actual field trials, it is very difficult to measure and control the input power of the athletes. Besides that, the modified oars may induce a different “feel” for the athletes that may compromise the outcome of such field trials. To avoid such complications one can revert to a robotic rowing boat (9,10). It was therefore decided to develop and employ a robotic rowing boat that can be equipped with different blade configurations. Then sensors can be used to measure the actual forces on the oars and the oar displacements. From this the actual work done for each stroke cycle is determined. In this study the blade surface area was varied, and the input power and boat speed are compared for various configurations in blade angle and surface area.

Grift et al. (1) observed that by tilting the blade the efficiency of the blade motion becomes optimal, i.e. by directing the impulse vector parallel to the boat velocity, while at the same time the propulsion effectiveness decreases; hence, making the blade more efficient at equal stroke rate and oar angle reach then results in a lower input power and also lower boat velocity. This can be understood as follows (11): Consider a conventional blade with 0-degree angle and a modified blade with a 15-degree angle. When rowing at equal stroke rate and oar angle reach, both blades have a (nearly) equal velocity U in the water. To move the 0-degree blade through the water with a velocity U, a force $F_{0}$ is required, and this gives the boat a speed $V_{0}$. For the 15-degree blade, a smaller force $F_{15}<F_{0}$ is applied at equal stroke rate and oar angle reach, i.e. the blade moves along the same path and with the same velocity in the water. Hence, the power input, given by the product of blade velocity and blade force, is lower: $F_{15}\times U<F_{0}\times U$. The input power for the propulsion is balanced by the boat drag force times it velocity, where the boat drag force D is proportional to the square of the boat speed V, i.e. $D∼V^{2}$, so that $V\times D∼V^{3}$. Since less propulsive power is provided by the 15-degree blade (at equal stroke rate and oar angle reach), also the boat speed is lower: given that $F_{15}<F_{0}$, it is found that $V_{15}<V_{0}$. It should be noted that for equal power input, a 15% more efficient blade only gives an increase of 5% in boat speed, i.e. $1.15^{1/3}=1.05$. In a similar vein, the more efficient blade would have a slightly lower velocity U through the water as one needs to correct U for the boat speed V when applying equal stroke rate and oar angle reach, while it was assumed that the blade velocities for both blades are equal; however, this is a second-order correction to the general analysis.

The principal aim of the work presented here is to investigate and demonstrate that the forward tilted rowing blade would result in a more efficient and effective motion of the blade through the water that would result in a higher boat speed when an equal input power is provided, as suggested by the findings of Grift et al. (1). Hence, to demonstrate that the optimal blade actually leads to a better performance, i.e. higher boat speed, it is necessary to compare boat speeds at equal power input. To achieve equal power input, one has to take either of three actions: (i) increase the blade speed by increasing the stroke rate, (ii) increase the blade surface area, or (iii) use a longer oar shaft. In the present study it was chosen to keep the same stroke rate and oar shaft lengths, and vary the blade area.

In Section “Experiment” the robotic rowboat and experiments are described. The results are presented in Section “Results,” and Section “Discussion” summarizes the findings of this investigation.

## Experiment

Since it is difficult to assess the rowing propulsion efficiency in field trials, a robotic 1:5 model of a rowing boat with two oars is developed where different blade angles can be tested, while monitoring and controlling the power input. The scaled model is intended to be used in a towing tank, which limits the dimensions. The robot can be equipped with different 3D-printed blades at various angles to the oars (see Figure 4). Although both stroke rate and oar angle reach of the driving phase, i.e. the oar angles for the catch and release (see Figure 3), can also be varied, it was decided to change only a single parameter, that is the blade area. Varying the area of the blades could be easily achieved by scaling the blade dimensions for the 3D printing of the blades. The 1:5 scale is chosen so that the rowing robot can be operated in a towing tank. This provides the possibility of accurate measurement of the boat speed and propulsion power. However, the use of a scaled model implies opposing effects in Reynolds number and Froude number; this is discussed in more detail below.

**Figure 2:**
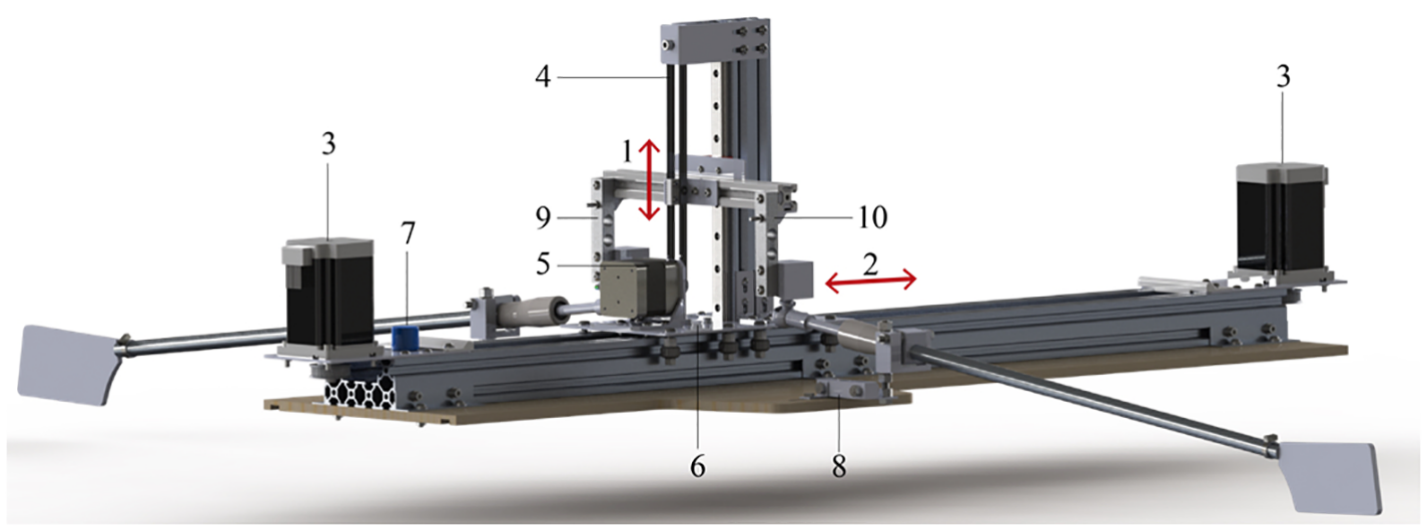
Schematic of the rowing robot: (1) vertical motion axis; (2) horizontal motion axis; (3) horizontal-axis motors (2×); (4) pulley belt system; (5) vertical-axis motor; (6) carriage; (7) potentiometer for horizontal displacement (replaced by an acoustic distance meter in the present measurements); (8) port side oar handle force sensor; (9) starboard oar handle force sensor. The boat hull is not shown. From: Keizer et al. (12).

A schematic of the robot is shown in Figure 2. Two stepper motors (ST6018L3008-A Nanotec with ACT DM542S drivers) use a belt drive to move a carriage along a horizontal slider, and an additional stepper motor uses a belt drive to impose the vertical motion. The robot is controlled with an Arduino UNO (RRID:SCR_017284). Strain gauges (half bridge configuration with scaime CPJ RAIL 120495 amplifier) are used to measure the actual forces applied to the oars, and the displacement of the horizontal carriage (item (6) in Figure 2) is measured by an ultrasound distance meter (Honeywell 940-R4Y-AD-1C0). This replaced the original potentiometer (see Figure 2), which gave a very noisy signal. From these data the actual work that is delivered in each stroke cycle can be inferred. The data are acquired using LabView in combination with a data acquisition system (NI USB-6211).

**Figure 3:**
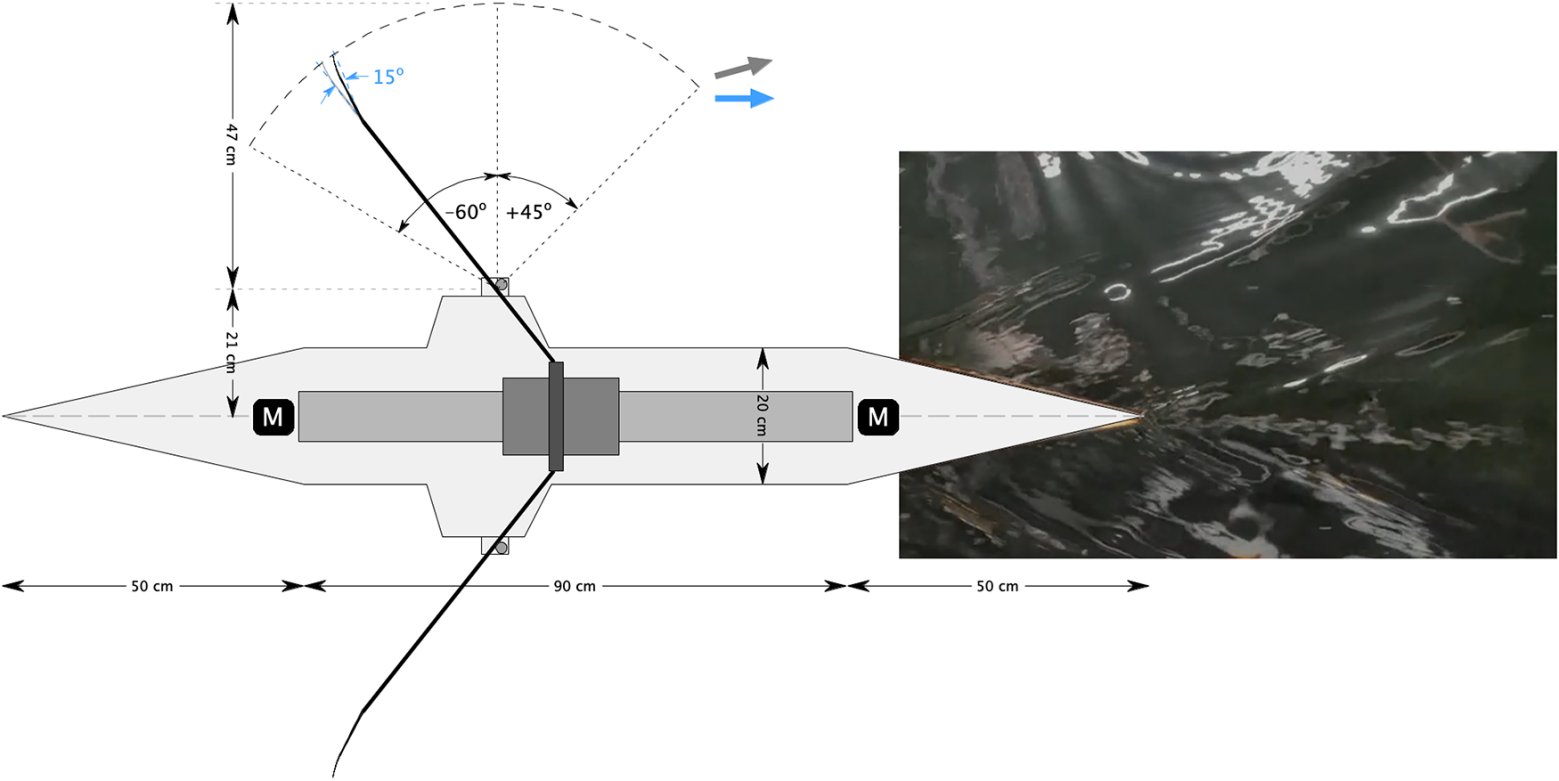
Schematic top view of the rowing robot, with dimensions and oar angles for the catch ($-60^{\circ }$) and release ($+45^{\circ }$), and the tilted blade ($+15^{\circ }$). The total length of the hull is 190 cm. For a conventional blade (0${}^{\circ }$ tilt) the eddies come off at an angle of about 15${}^{\circ }$ with respect to the direction the boat is moving (gray arrow); for the blade with a 15${}^{\circ }$ tilt the eddies move parallel to the boat (blue arrow). Inset shows a photograph of the wave pattern at the stern.

**Figure 4:**
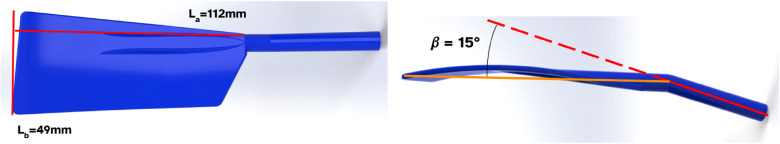
(left) Front view of the 3D-printed 1:5 scaled model of a “Big blade” used in this research with the 100% reference blade area; (right) Top view of the blade with a $15^{\circ }$ forward angle with respect to the oar (at 100% reference blade area).

In this study, 3D printed 1:5 scaled “Big blade” blades are used; see Figure 4. The optimal blade angle ($\beta =15^{\circ }$) is tested against the conventional blade configuration ($\beta =0^{\circ }$). To vary the work of the blade delivered to the water the blade surface area of the tilted blade is varied between 100% and 110%, with 2% increments, of its original area. The motion of the blades mimics the actual motion of rowing blades obtained from field measurements, with an angle of $-60^{\circ }$ for the catch, and $+45^{\circ }$ for the release (see Figure 3), where $0^{\circ }$ is when the oar is normal to the boat (1,13). The blades gradually accelerate to achieve a smooth entrance and release of the blades through the water. The blade pitch remains at $0^{\circ }$ during the entire rowing cycle. In all measurements the stroke rate is set to 25.5 min${}^{-1}$, i.e. a cycle period of 2.347 sec. This is lower than the typical stroke rate of 36–40 min${}^{-1}$ during a race. However, one must take into account the model scale of the oars and boat. For the model blades and robot, the Strouhal number, St=fL/V, (14) where f is a frequency (in [1/s]), L a typical length scale, and V a typical velocity, is estimated at 0.069, based on the stroke rate, blade length and speed; this value for the Strouhal number is close to the value of 0.11 for the full scale situation rowing at race pace (1). Furthermore, there are technical limitations, as the chance for drive belt failure increases with stroke rate, which was why a stroke rate of 25.5 min${}^{-1}$ was used in an earlier version of the robot (12). The Reynolds number (Re) and Froude number (Fr) of the scaled blades are Re $∼77\times \;10^{3}$ and Fr≅1.0, respectively, while the full size blades would be characterized by Re∼110-330×103 and Fr∼0.34-1.02, respectively. It can be safely assumed that the Reynolds number is sufficiently high where the drag and lift coefficients of the blade become independent of the blade Reynolds number, and the Froude number of the scaled blades is within the range of the full-scale blades, so that there exists a similar influence of the air-water surface (1,15). During the whole cycle the blades maintain their vertical orientation; in actual rowing the blade pitch is changed during the recovery stroke (8). This would have complicated the design and construction of the rowing robot, and was not considered essential for the present purpose of the experiments. A summary of the blade characteristics is presented in Table 1.

**Table 1:** Summary of the characteristics of the rowing blade, rowing boat and towing tank. (The symbol g=9.8 m/s${}^{2}$ represents the gravitational acceleration, and $\nu =1.0\times 10^{-6}$ m${}^{2}$/s the kinematic viscosity of water.)

Rowing blades:		
Length ($L_{a}$)	112	mm
Height ($L_{b}$)	49	mm
Stroke rate (f×60)	25.5	[min${}^{-1}$]
Typical velocity (U)	70	cm/s
Strouhal number ($\mathrm{St}=fL_{a}/U$)	0.069	[–]
Reynolds number (Re=ULa/ν)	$77\times 10^{3}$	[–]
Froude number ($\mathrm{Fr}=U/\sqrt{gL_{b}}$)	1.0	[–]
Hull:		
Length (ℓ)	190	cm
Width	20	cm
Draft	8.3	cm
Maximum transverse cross section ($A_{X}$)	123	cm${}^{2}$
Wetted area	0.46	m${}^{2}$
Typical velocity ($V_{0}$)	78	cm/s
Froude number ($\mathrm{Fr}=V_{0}/\sqrt{g\ell }$)	0.18	[–]
Drag coefficient ($C_{D}$)	0.016	[–]
Towing tank:		
Length	220	m
Width (W)	4	m
Depth (H)	3.6	m
Blockage ratio ($m=A_{X}/W\times H$)	<0.1	[%]
Depth Froude number (${\mathrm{Fr}}_{H}=V_{0}/\sqrt{gH}$)	0.13	[–]
Blockage velocity correction${}^{a}$	<0.1	[%]
Wave resistance velocity correction${}^{a}$	0.00	[%]

${}^{a}$Schuster’s method in ITTC report 7.5-02-02-01 Rev. 3 (2011).

**Figure 5:**
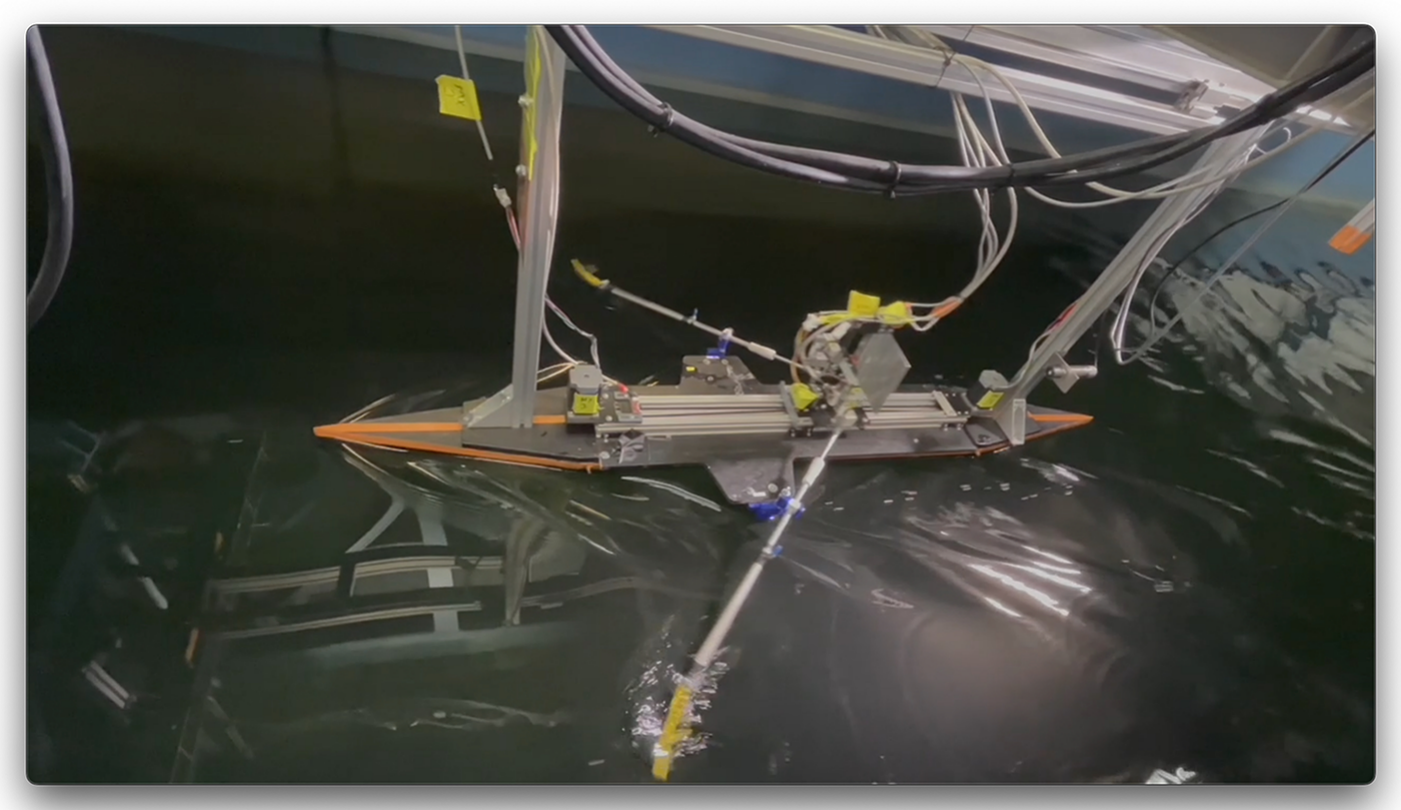
The rowing robot mounted under the carriage of the MARIN Concept Basin towing tank. See also the video in the Supplementary Material.

The robot is fitted on a 190 cm length round hull, with tapered bow and stern; see Figure 3. The use of a 1:5 model hull has implications of the Froude number, $\mathrm{Fr}=V/\sqrt{g\ell }$, where V is the boat speed, g the gravitational acceleration, and ℓ the boat length, which is about 0.18 for the model boat. This is considerably lower than the typical value of Fr=0.5-0.6 for full-scale rowing boats, and mainly has implications for the drag force (16). As mentioned previously, the principal aim of this study is to investigate and demonstrate the effect of tilting the rowing blade; for this purpose, it was not considered essential to have a realistic model of the hull, and an approximate slender hull would be sufficient for the present purpose. The round hull has a radius of 10 cm. The total weight of the robot and hull is 13 kg, and the mass that participates in the oscillating motion to drive the oars is estimated at 1 kg. It is noted that this is not realistic with respect to the ratio of boat mass versus oscillating mass in an actual rowing boat. Also, because of the weight, the boat cannot float by itself, and therefore is suspended from a sliding rail mounted to the towing tank carriage; see Figure 5. The boat draft is then set to 8.3 cm, and the maximum transverse cross section $A_{X}$ of the model hull is 123 cm${}^{2}$. The total wetted area is 0.46 m${}^{2}$, and the boat hull drag coefficient $C_{D}$ is estimated at 0.016, which is given by $C_{D}=D/(\frac{1}{2}\rho V^{2}A)$, where D is the drag force, ρ the density of water, V the mean boat velocity, and A the wetted area of the hull (14). The value for $C_{D}$ is found from equating the input power P=2W/T, where W is the work per stroke (and per oar) and T the duration of the stroke cycle, to the work performed by the drag per unit of time, given by D×V. Please note that this estimate does account for small mechanical losses in the power estimate, so it should be considered as an upper limit. Yet, this drag coefficient is not optimal and likely higher than that of an actual rowing boat (16,17), but as mentioned before, in this study it was not the objective in this study to accurately represent the hull of an actual rowing boat. As a matter of fact, a more representative hull shape would probably have led to a much smaller force to drive the oars, which then is more difficult to measure accurately in the current configuration. A summary of the hull characteristics is presented in Table 1.

Tests were carried out in the Concept Basin at MARIN, which has a length of 220 m, a width of 4 m and a depth of 3.6 m.^1^ A summary of the towing tank characteristics is presented in Table 1, including relevant parameters related to blockage and wave drag corrections following the recommended procedures and guidelines of the International Towing Tank Conference (ITTC). The boat is mounted on a sliding rail from the carriage of the towing tank; see Figure 5. This allows free forward and backward motion of the rowing boat, as it accelerates and decelerates during each stroke. The towing tank carriage follows the boat at a constant speed, while the position of the robot relative to the carriage is measured using a second ultrasound distance meter (PIL P42-T4V-2D-1C0-130E) that measures the position of the rowing robot with respect to a reference plate that is mounted on the carriage. A few trial runs were done prior to each set of measurements to find the proper speed of the towing tank carriage for each blade configuration. Occasionally, minor adjustments were made to the towing tank carriage speed if it appeared that the rowing boat was advancing or receding with respect to the towing tank carriage. A single run in the towing tank would take around 165 sec, which is sufficient to record 60–70 strokes at a stroke rate of 25.5 min${}^{-1}$. The towing tank carriage first accelerates from rest to the target velocity in about 8 sec. It then takes another 3–5 strokes before the rowing boat reaches a steady back-and-forth motion with a mean speed that is equal to the towing tank carriage speed. At least 5 measurement runs were taken for each blade configuration, with a total of 12 different blade configurations. For the analysis of the measurement data 20 strokes are taken from each run that occur after 20 sec from the moment the towing tank carriage begins to accelerate. This allows enough time to reach a steady rowing motion. During each run, the data for the speed of the towing tank carriage, the position of the rowing boat relative to the towing tank carriage measured with the ultrasound distance meter, the position of the rowing boat carriage that drives the oars measured with the ultrasound distance meter, and the forces on the port-side and starboard oars were measured and collected using the data acquisition system. The data recording rate was 33 Hz.

## Results

**Figure 6:**
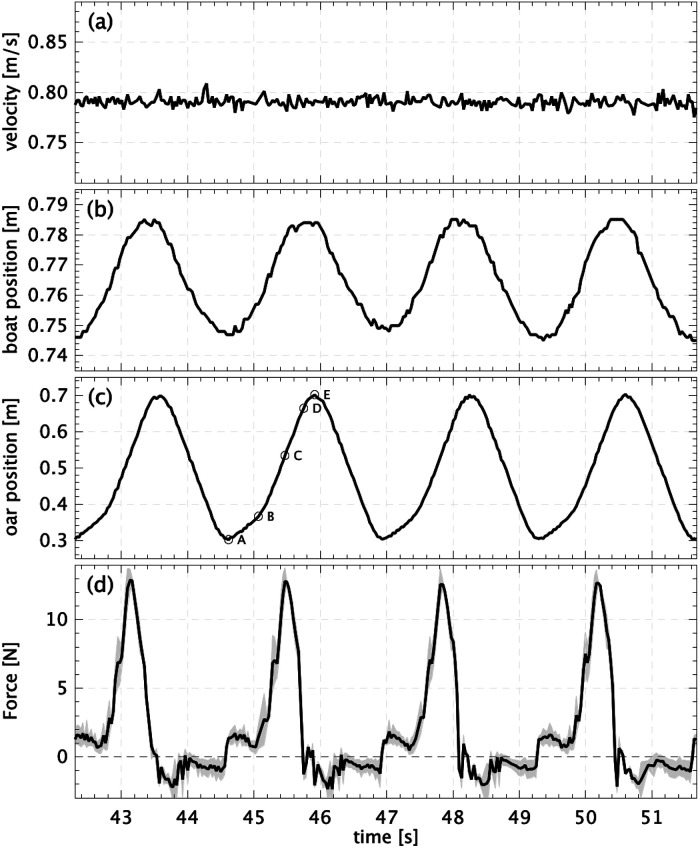
Example of measured signals during four strokes of the 0-degree blade with 106% surface area. (**A**) The velocity of the towing tank carriage; (**B**) the position of the boat relative to the carriage; (**C**) the position of boat carriage that drives the oars. Labels indicate the start (A) and end (B) of the catch, position when force is maximal (C), and the start (D) and end (E) of the release. (**D**) The measured force (averaged over both oars); the gray region indicates the variation between the forces on the port-side and starboard oars.

An example of the measured signals during four rowing strokes of the 0-degree blade with 106% surface area is shown in Figure 6. In this example the towing tank carriage speed is nearly constant at 0.79 m/s; see Figure 6A. The rowing boat moves back-and-forth relative to the towing tank carriage over a distance of 4–5 cm (Figure 6B). During a stroke the relative velocity of the rowing boat is about −55 mm/s during the drive, and about +40 mm/s during the recovery. Hence, the relative variation in velocity is approximately 12% of the mean boat speed. This is smaller than the 20–25% variation in actual rowing (18) and the 30% in the work of Labbé et al. (10), and can be attributed to the relatively large mass of the boat with respect to the oscillating mass in the boat that is driving the oars.

Figure 6C shows the horizontal position of the carriage that drives the oars. The label (A) indicates the start of the catch when the blades begin to drop vertically into the water. This is at an angle of −60${}^{\circ }$ with respect to the boat normal; see Figure 3. The blades advance gradually till (B). This is to avoid any sudden impulsive motion due to the large difference in velocity between the water and the blades. Then the oars accelerate to a higher velocity. At (C) the largest force is measured when the oars are approximately normal to the boat ($0^{\circ }$ angle). The oar angle continues to increase to about $+45^{\circ }$, where the motion quickly decelerates and the blades are lifted from the water (E). The measured forces are shown in Figure 6D. The black solid line represents the average of the forces measured on both oars. There is a slight random difference in the forces measured on the port side and starboard oars; this is indicated by the gray band. Differences between the measured forces possibly originate from measurement noise in the two force sensors, but may also be due to small variations in model blade manufacturing, mounting precision, mechanical play in the robot mechanism, or cycle-to-cycle variation in the depth of the blades below the water surface. However, the mean absolute difference is between ±0.3 and ±0.9 N, averaged over each run, which is 2–7% of the maximum measured force during a cycle.

The measured velocities of the towing tank carriage show a variation of less than 2% (root-mean-square value) for each run (see Figure 6), giving an error of 0.1% for estimated mean (95% reliability margin). However, the values for the velocity showed a wider spread between the five runs for each case; the difference between the maximum and minimum mean velocities is found to be between 0.0 and 1.6% of the mean velocity averaged over the five runs. It should be noted here that the Concept Basin towing tank carriage is intended to operate at much higher speeds, up to 10 m/s, and the variation in speed of 0.012 m/s is taken as a variation in reproducing the same (low) velocity for the towing tank carriage.

Mechanical work is defined as the integral of force times displacement (19). The total work W (in Joule) per stroke is determined by integrating the measured force F over the displacement s of the oar carriage, i.e.

(1) $$W=\frac{1}{N}\int_{t_{1}}^{t_{2}}F\;ds,$$

with: $t_{2}-t_{1}=N\cdot T$, where N is the number of strokes, and T the stroke period. The integral (1) is computed by simple quadrature, i.e. $W=\sum_{i}F_{i}\Delta s_{i}/N$, with: $\Delta s_{i}=s_{i}-s_{i-1}$. It is noted that the entire motion is considered, including the recovery, as this would also be part of the power needed in rowing, although the actual contribution to the total power is very small (between 7 and 10%) compared to that of the drive motion.

The measurement error in the work per stroke is estimated by taking the standard deviation of the work for each of the 20 cycles. The root-mean-square cycle-to-cycle variation is between 0.6% and 4.0%, with an average of 1.3%, for the five runs over the different cases; this gives an estimated error of less than 1.8% (95% reliability interval) for the measured average work per stroke during each run. Also here there is some variation between the average results over the different runs where the absolute differences between the maximum and minimum mean values for work per stroke over the five runs per case are between ±0.3% and ±2.4%, i.e. in the same range as the estimated statistical error for the mean work per stroke in the measurements.

**Figure 7:**
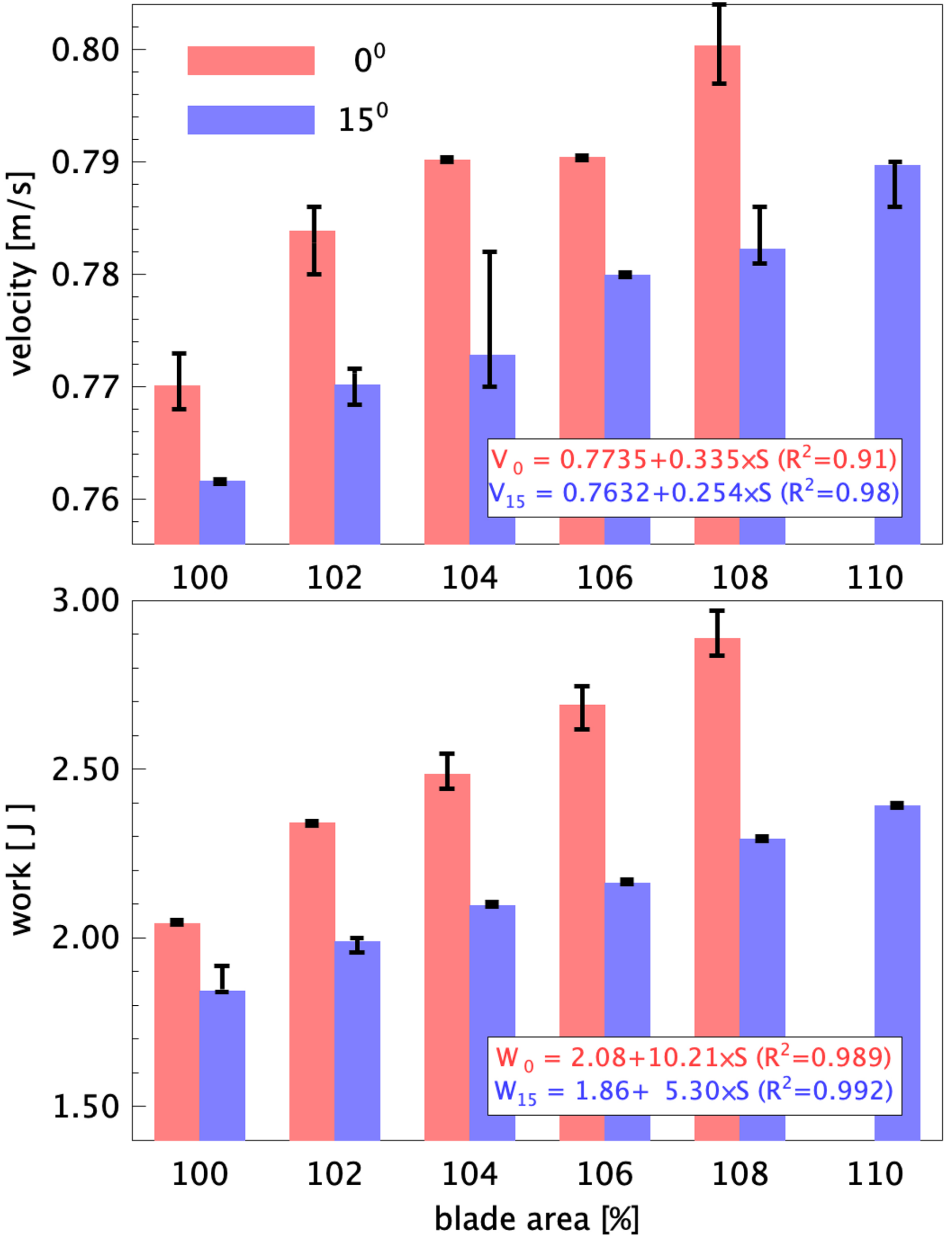
Bar graphs of the mean boat velocity (top) and the work per stroke (bottom) for the 0 and 15${}^{\circ }$ tilted blades for the original blade area (100%) and increased blade areas between 102 and 110%. The data for the 0${}^{\circ }$ blade with 110% surface area are excluded. Error bars indicate the maximum and minimum values for the measured averages during the runs; insets give the linear fits as a function of relative increase in blade area (i.e., 106% blade area corresponds to S=0.06) with correlation of determination (R${}^{2}$) between parenthesis.

The measured boat velocity and work per stroke for each blade configuration are presented in Figure 7. It is noted that the results for the 0${}^{\circ }$ blade with 110% surface area are excluded, as it appeared that for this case the rowing boat was towed by the towing tank carriage through the electrical and data cables, which compromised the measurement results. It can be seen that both the boat speed and work increase for increasing blade area for both 0 and 15${}^{\circ }$ configurations. Note that all measurements are done at the same stroke rate; hence, for the more efficient configuration with 15${}^{\circ }$ tilt the results for the speed and work are always lower than those of the conventional configuration with 0${}^{\circ }$ tilt of the blade. The difference in velocity between the 0 and 15${}^{\circ }$ configurations appears to be more or less constant at 0.1 to 0.18 m/s for cases with equal blade surface area. The work per stroke increases directly proportional with increased blade surface area, and it can be seen that the rate of increase for the 15${}^{\circ }$ blade with blade surface area is at a lower rate than for the 0${}^{\circ }$ blade. This is already a clear indication that the 15${}^{\circ }$ blade is a more efficient configuration, i.e. the gain in speed by using a larger blade requires a smaller increase in work by the athlete.

**Figure 8:**
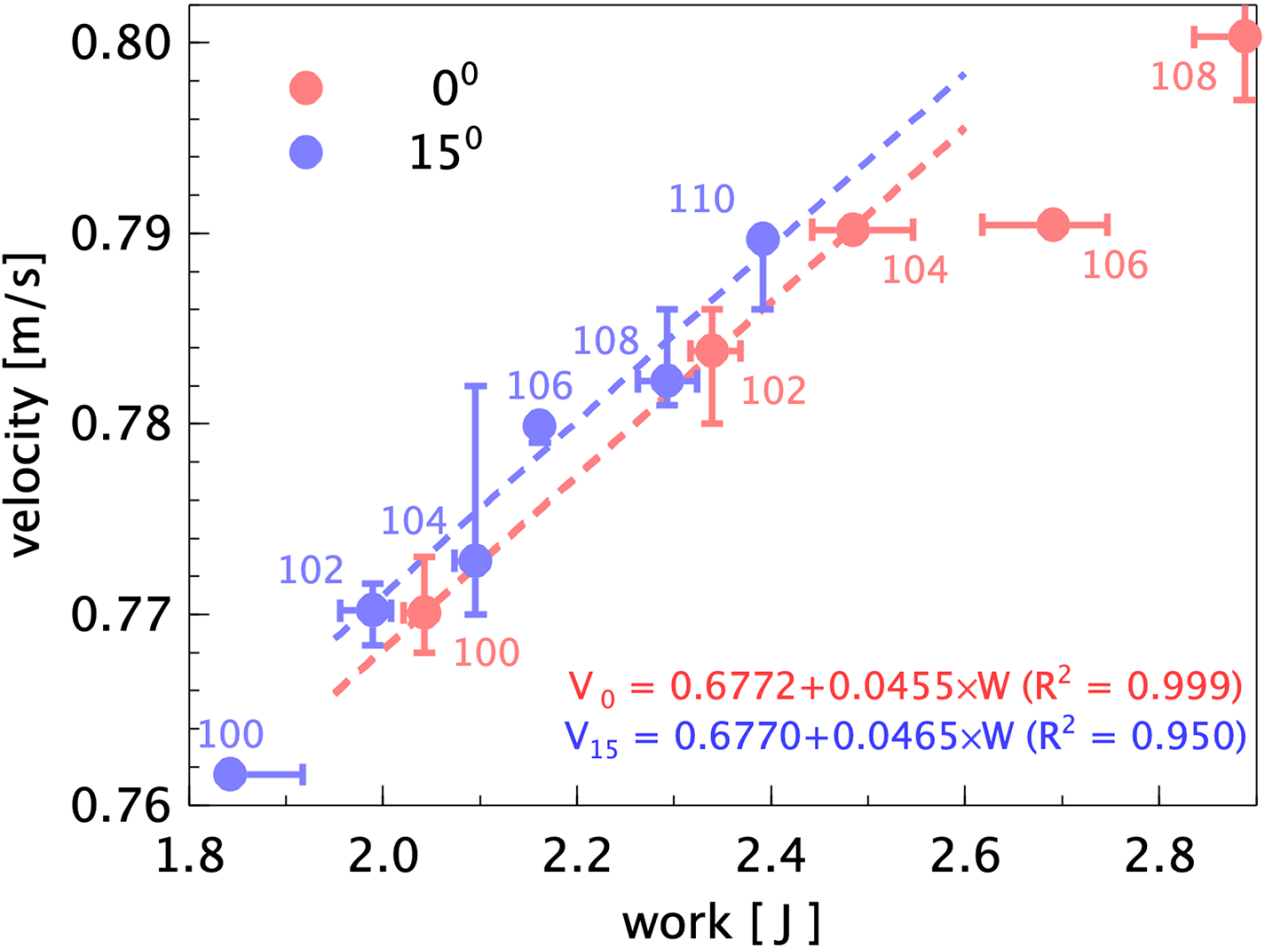
The measured mean boat velocity as a function of the input work per stroke for the 0 and 15${}^{\circ }$ blade configurations. The labels indicate the blade surface area in % relative to the standard blade. Error bars indicate the maximum and minimum values for the measured averages during the runs; insets give the linear fits of the velocity as a function of input work per stroke, with correlation of determination ($R^{2}$) between parenthesis.

In order to quantify the improvement in performance, the boat velocities need to be compared for equal input work per stroke. This is presented in Figure 8. In this graph the data from Figure 7 is plotted, and fitted lines through the data points between values of 1.9 and 2.6 J for the work per stroke. It is noted that the data points for the 15${}^{\circ }$ tilted blades have some larger variation in the boat velocity, which can be attributed to the finite accuracy by which the speed of the towing tank carriage can be matched to the mean rowing boat speed during the measurement campaign. The data points for the 106% and 108% 0-degree blades appear to deviate from the other data; it is suspected that for these cases the power and data cables between the robot and the towing tank carriage may have dragged on the boat, leading to a higher power input and a lower boat velocity. However, it appears that the two lines could be fitted with nearly identical slopes over the given range, i.e. there is a constant difference in velocity of 0.3 cm/s for equal input power, in favor of the 15${}^{\circ }$ tilted rowing blades, while all data points for the 0-degree blade lie below the fitted curve for the 15-degree blade. This implies a 0.4% higher speed at equal power input. Although this difference may be small, it would translate to reduction of 1.53 sec with respect to the 6:33.26 time, set in 2014; this then corresponds to a length of almost 8 m at the 2 km finish line.

**Figure 9:**
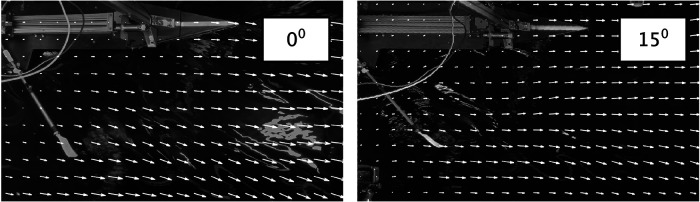
Motion of surface flow patterns for the 0 and 15${}^{\circ }$ blades. The camera is moving along with the towing tank carriage. Arrows indicate the motion of surface patterns in a video sequence, overlaid on a still frame near the blade release.

Finally, quantitative visualizations are performed of the surface flow patterns generated by the blades moving through the water. The blades displace the water and generate eddies that deform the water surface. This is visible from the ambient light that is reflected from the water surface. The undisturbed surface water remains at equal light intensity, while the disturbed water is characterized by a varying light intensity that moves with the flow. Video sequences are taken, and the intensity difference between each pair of subsequent frames is computed. This results in visualizing only the fluctuating light intensity of the water surface reflections, while suppressing the constant background. Then an image correlation analysis is performed by means of particle image velocimetry (20) in order to determine the motion of the water surface patterns. The results of this analysis are shown in Figure 9. This shows that the eddies that are generated with the 0${}^{\circ }$ blade make a larger angle with respect to the direction of motion of the rowing boat than for the 15${}^{\circ }$ blade. The motion normal to that of the rowing boat implies a loss of propulsion efficiency, as demonstrated by Grift et al. (1).

## Discussion

The aim of the present investigation is to validate the results of the laboratory investigation that a rowing blade tilted by $15^{\circ }$ would improve the propulsion efficiency in rowing by avoiding losses as these tiled blades displace water in a direction more parallel to the boat velocity, as suggested by Grift et al. (1). To avoid difficulties anticipated in field studies, a robot rowing boat is built where the input power and boat velocity can be measured. This rowing robot is constructed at a 1:5 scale, so that tests can be done in a towing tank under controlled conditions. Evidently, a scaled rowing boat implies that one needs to compromise in term of the flow conditions, characterised by the Reynolds, Froude, and Strouhal numbers. As mentioned in the Introduction, the decrease of the effectiveness by increasing the blade angle was compensated for by increasing the size of the blade such that it was possible to compare the resulting boat velocities at equal power input. Alternatively, this could also have been compensated by increasing the stroke rate or the oar length. The present rowing robot appears to produce realistic time histories of the forces that act on the oars (see Figure 6), and the visualizations in Figure 9 show that the eddies that are generated with the 0${}^{\circ }$ blade are oriented at a larger angle with respect to the boat velocity than those generated with the 15${}^{\circ }$ blade. The final results (Figures 7, 8) appear to show that, at equal power input, the 15${}^{\circ }$ tilted blades make the boat go at a slightly higher speed, but that could be large enough to make a difference at the finish line. However, there is room for improvement for the hull shape and rowing dynamics, i.e. the oscillating mass relative to the boat mass. However, the largest improvements in the experiment can be made by re-designing the way in which electrical power is supplied and data is transferred to and from the rowing boat. In the current setup thin electrical cables are used, but this does not avoid that these cables can pull or drag the boat, which affects the measurement results; as a result of this, certain data points clearly deviated from the other data, and were excluded in the further analysis. To fully eliminate this effect it is considered to switch to the use of on-board batteries for the electrical power supply, and wireless data transfer through Bluetooth technology. Nonetheless, the usefulness of an instrumented robot rowing boat as an intermediate step between laboratory research and field measurements has been demonstrated.

From the current measurements it can be concluded that (i) there appears to be an improvement in boat speed using tilted rowing blades with equal power input as compared to conventional non-tilted rowing blades (Figure 8). The measurements show that (ii) increasing the boat speed by increasing blade area requires a smaller increase in input power using the 15${}^{\circ }$ blades than using conventional blades with a 0${}^{\circ }$ tilt of the blades (Figure 7). (iii) The 15${}^{\circ }$ tilted blades also appear to avoid propulsion losses as they displace water in a direction more parallel to the motion of the rowing boat, as suggested by Grift et al. (1); this was validated in the flow visualization results presented in Figure 9. This makes the alternative 15${}^{\circ }$ configuration of interest in finding ways of improving rowing performance. (iv) From the results presented in Figures 7 and 8 a configuration that combines the 15${}^{\circ }$ blade angle with a 104% or 106% blade area is recommended.^2^

Further improvements in optimizing the shape and configuration of the rowing blade, such as for example the blade angle, area, aspect ratio, and camber, are anticipated by using the hydrodynamic insights on the contributions of drag and lift to the propulsion provided by the experiments of Grift et al. (1). Future research will also investigate the effects of changing stroke frequency and varying the oar length. Although the present rowing robot appears to give realistic time histories of the forces on the oars (Figure 6), the hull shape and rowing dynamics, i.e. the oscillating mass relative to the boat mass, could be improved. Parallel to this investigation, field tests are carried out to explore the introduction of this innovation in competitive rowing.

## Conclusion

The outcome of this study supports that a rowing blade with a 15${}^{\circ }$ forward angle with respect to the oar shaft can have a positive impact on rowing performance. The modified blade results in a more efficient and effective motion of the blade through the water (1). At equal stroke rate, the blade area is increased to 4–6% to yield the same input power, which results in a 0.4% higher boat speed.

## Data Availability

The raw data supporting the conclusions of this article will be made available by the authors, without undue reservation.

## Footnotes

^1^ www.marin.nl/en/media-centre/leaflets-facilities#basins

^2^Or alternatively, an equivalent increase in stroke rate or oar length.
